# Characterising smoking cessation smartphone applications in terms of behaviour change techniques, engagement and ease-of-use features

**DOI:** 10.1007/s13142-015-0352-x

**Published:** 2015-11-23

**Authors:** Harveen Kaur Ubhi, Susan Michie, Daniel Kotz, Onno C. P. van Schayck, Abiram Selladurai, Robert West

**Affiliations:** 1 Cancer Research UK Health Behaviour Research Centre, University College London, London, WC1E 6BT UK; 2 Department of Family Medicine, CAPHRI School for Public Health and Primary Care, Maastricht University Medical Centre, Maastricht, The Netherlands; 3 Centre for Outcomes Research and Effectiveness, Research Department of Clinical, Educational and Health Psychology, University College London, London, UK; 4 Institute of General Practice, Medical Faculty of the Heinrich-Heine-University Düsseldorf, Düsseldorf, Germany

**Keywords:** Smoking, Smoking cessation, Smartphone, Apps, Applications, Mobile, Behaviour change interventions, Behaviour change techniques, Taxonomy, BCTs, Engagement, Ease-of-use, Feature

## Abstract

The aim of this study was to assess whether or not behaviour change techniques (BCTs) as well as engagement and ease-of-use features used in smartphone applications (apps) to aid smoking cessation can be identified reliably. Apps were coded for presence of potentially effective BCTs, and engagement and ease-of-use features. Inter-rater reliability for this coding was assessed. Inter-rater agreement for identifying presence of potentially effective BCTs ranged from 66.8 to 95.1 % with ‘prevalence and bias adjusted kappas’ (PABAK) ranging from 0.35 to 0.90 (*p* < 0.001). The intra-class correlation coefficients between the two coders for scores denoting the proportions of (a) a set of engagement features and (b) a set of ease-of-use features, which were included, were 0.77 and 0.75, respectively (*p* < 0.001). Prevalence estimates for BCTs ranged from <10 % for medication advice to >50 % for rewarding abstinence. The average proportions of specified engagement and ease-of-use features included in the apps were 69 and 83 %, respectively. The study found that it is possible to identify potentially effective BCTs, and engagement and ease-of-use features in smoking cessation apps with fair to high inter-rater reliability.

**Electronic supplementary material:**

The online version of this article (doi:10.1007/s13142-015-0352-x) contains supplementary material, which is available to authorized users.

## INTRODUCTION

There are a large of number of smartphone applications (hereon referred to as ‘apps’) available that purport to aid smoking cessation. It is not feasible to undertake randomised controlled trials (RCTs) to evaluate all of these, or even the most popular ones. This raises the question as to how these apps may be evaluated. One way is to identify specific features that would be expected to lead apps to become effective, engaging and easy to use. This paper reports a study, which sought to address that gap in the literature. If a reliable system to code app features is found, then future studies can use this approach to correlate app features with the usage and outcome data.

An estimated 1.4 billion or 20 % of the world’s population owns a smartphone [1]. Smartphones can deliver a range of behaviour change interventions because of their ability to run programs known as apps, which can be tailored to users’ needs and are available when needed [2, 3]. Currently, there are more than 28,000 ‘medical’ apps available on two major platforms, iOS and Android [4]. ‘Medical’ apps include a subset of apps that are known as ‘health and fitness’ apps. ‘Health and fitness’ apps focus on various aspects of health including helping people adopt healthier behaviour patterns. These apps are becoming increasingly popular [5]. In the USA alone, there were approximately 18.5 million ‘health and fitness’ app users in 2011 [6]. Around 400 apps are aimed at helping smokers to quit, but very little is known about their content [7–11] or effectiveness [12–14].

For most smokers, smoking cessation is the single-most important change they can make to their behaviour to improve their life expectancy [15] and quality of life [16]. A recent study found that more than 700,000 downloads of smoking cessation apps are made by Android users every month [17]. In another recent study, almost half of smokers had used an app to support their quit attempt [18]. If such apps support behaviour change, they could confer a considerable benefit to public health. On the other hand, if they are ineffective, then at the very least, this represents a wasted opportunity. It is important that every attempt a smoker makes to quit should have the highest likelihood of success given the negative consequences of a failed quit attempt. Every year of continued smoking costs an average of 3–6 months of life expectancy [19]. It would be unrealistic to expect that more than a tiny fraction of smoking cessation apps would ever be evaluated in RCTs, and yet, there is a need to gauge how effective such apps are likely to be.

One way to evaluate apps is by assessing whether or not smoking cessation apps provide the advice, information or activities that have been shown with other modes of delivery to aid smoking cessation. This has been attempted in recent studies [7, 8]. Smoking cessation apps were evaluated using two criteria: (1) the apps’ general approach and (2) their score on an index based on conformity with the US Public Health Service’s Clinical Practice Guideline for Treating Tobacco Use and Dependence [20]. It was found that the conformity with the guideline was low. No apps recommended calling a quitline, while only a handful recommended using licensed stop-smoking medication. Another study attempted to evaluate smoking cessation apps using the precepts of Self Determination Theory [9]. The theory argues that humans have three basic needs beyond our biological drives: autonomy, competence and relatedness [21]. The evaluation concluded that smoking cessation apps may not sufficiently stimulate autonomous motivation and only a small number of apps addressed all three basic needs [9]. A further study attempted to assess Facebook apps for smoking cessation (Facebook apps are available on personal computers and some are also accessible on mobile and/or smartphones) [10] on two criteria: (1) content features (interactive, informational and social) and (2) their score on an index based on conformity with the US Public Health Service’s Clinical Practice Guideline for Treating Tobacco Use and Dependence [20]. The study found low conformity with the guideline, and only three out of nine apps recommended treatment components [10]. Another recently published study conducted a content analysis of Android smoking cessation apps [11]. The study recorded app popularity and user-rated quality (based on the number of stars awarded by the users) from Google Play store and coded the existence of tailoring features in the apps within the context of using the ‘5 A’ (‘ask,’ ‘advise,’ ‘assess,’ ‘assist’ and ‘arrange follow-up’), as recommended by the US Public Health Service’s Clinical Practice Guideline for Treating Tobacco Use and Dependence [20]. The study found that Android smoking cessation apps were not ‘smart,’ and the apps fall short of providing tailored feedback. However, 18 % (41 out of 225 apps) of the Android smoking cessation apps were found to discuss pharmaceutical products. The above studies provide useful information but lack specificity in terms of coding what may be expected to be ‘active’ ingredients (i.e. ingredients that can actually contribute to behaviour change) of apps and do not allow comparisons between smoking and other target behaviours in a way that could inform understanding of behaviour change mechanisms. A complementary approach is to assess the likely effectiveness of apps in terms of use of specific ‘behaviour change techniques’ (BCTs) [22] associated with higher success rates in face-to-face behavioural support for smoking cessation. BCTs provide a way of characterising the content of behaviour change interventions using a language that is consistent across interventions and behaviours. BCTs also provide a means of assessing how behaviour change interventions link with particular theories and provide a basis for testing these theories [23]. A number of taxonomies of BCTs, including generic taxonomies of BCTs [22, 24] and those relating to specific behaviours such as alcohol reduction [25], promoting physical activity and healthy eating [26], increasing safe sexual behaviours [27] and smoking cessation [28, 29] have been developed.

The study reported in this paper used an adaptation of a previously developed smoking-specific taxonomy to assess the use of BCTs in smoking cessation apps [28]. The previous taxonomy was developed using treatment manuals for interventions evaluated in clinical trials as well as guidance documents and smoking cessation treatment manuals used by the English Stop Smoking Services (SSS) in the UK. The English SSS constitute a national programme of support for cessation, offering free behavioural support and stop-smoking medication to all smokers in the country [30]. The service is paid for out of general taxation and has been in operation since 1998. In 2014, the English SSS treated more than 600,000 smokers. The services are configured in approximately 150 localities, which operate under general guidance but have freedom to develop their own programmes based on this guidance. This leads to considerable variability in delivery and outcomes, thus providing a natural experiment to test for the effectiveness of specific components. When the smoking-specific taxonomy [28] was applied to the treatment manuals used by each service, the authors found that it was possible to identify a subset of BCTs, which are associated with higher success rates in quitting smoking (i.e. a subset of most effective BCTs emerged) [31]. The BCTs found to be associated with higher success rates are [31] (1) strengthening ex-smoker identity, (2) providing rewards (usually praise) contingent on stopping successfully, (3) measuring expired-air carbon monoxide concentrations, (4) advising on changing routines, (5) advising and assisting with ways of coping with urges to smoke, (6) asking about use of stop-smoking medication, (7) advising on use of stop-smoking medication, (8) giving options for additional support and (9) eliciting clients’ views. All of these except for BCTs (3), (8) and (9) can be implemented on a smartphone app without additional add-on devices. Thus, the apps in this study were assessed on five specific BCTs (we combined the medication-related BCTs 6 and 7 into one BCT) that had previously been found to be associated with higher success rates in quitting smoking and could be implemented on a smartphone app. BCTs that relied on external accessories, which are not routinely available (e.g. use of an expired-air carbon monoxide monitor) and BCTs that relied on availability of the support (e.g. telephone support), which might not be available or might involve the need to respond flexibly to issues raised by users of the app, were not included in the evaluation.

BCTs relate to the content of interventions. However, the way that this content is delivered is also important for intervention effectiveness. Two types of app features would be expected to be important in ‘delivery’ of the content: (a) features promoting engagement and (b) features that make the app easy to use.

There is extensive literature available on user engagement in digital design, but most of the literature relates to products that serve an immediate function (e.g. booking a holiday) or for products that are used for leisure (e.g. watching a film). There is much less literature available on user engagement for ‘health and fitness’ apps and even less literature available for smoking cessation apps in particular. We searched online databases, reviewed key textbooks, blogs and e-magazines and consulted with experienced app developers to arrive at a set of features that we judged would possibly promote user engagement and make apps easy to use [32–36]. The set of engagement features is found in Table 1, and the set of ease-of-use features is found in Table 2.

**Table 1:** Features that could promote engagement

	Feature	Brief description
1	Personas and personification	Establish a ‘rapport’ between the smoker and personification of the app (e.g. by creating a visual sense of the team)
2	Transparency and realistic expectations	Set up clear expectations concerning how the app will be used early on
3	Shaping	Keep demands of the smoker to a minimum
4	Instant feedback/gratification/gamification (scoreboards, points, badges, leader-boards, achievements, assignments, etc.)	Engage users by providing instant feedback loops (provide user progression statistics). Always provide users with a rewarding experience when they visit the app (rewards motivate people for more rewards)
5	Visual cues and dashboards	Where possible use images (photos, graphics or videos) to convey information
6	Design for curiosity	Present new information each time the app is accessed
7	Personalisation	Promote engagement by using text messaging and emails
8	Autonomy	Give control, choice and personal relevance by asking questions
9	Personalized recommendations	Make app as interactive as possible—ask relevant questions, tailored feedback, videos, audio, gallery, emails, text messaging, etc.
10	App’s design and user interface	The app must look professional
11	Sequencing and design for reducing each session time	Structure sections (break complex tasks into small steps) and keep login sessions brief (each session should not take more than 5 min of the users’ time)

**Table 2:** Features that could enhance ease-of-use

	Feature	Brief description
1	Pattern recognition	Make use of the app as habitual as possible in terms of the location of different elements
2	Aesthetics	Keep main pages as simple and visually appealing as possible but encourage and make it easy to use
3	Minimum text	Keep text as brief as possible
4	Text formatting	Try to avoid grouping more than two sentences together, use plenty of headings, keep paragraphs short and use bulleted lists and highlight key terms
5	Page names	Navigation must be consistent and straightforward. Every page needs a name, the name needs to be in the right place (in the visual hierarchy of the page, the page name should appear to be framing the content that is unique to this page), the name needs to be prominent (combination of size, colour and typeface), the name needs to match with what user clicked
6	Easy-to-read	Reading level to age 14
7	Layout	Layout pages to avoid scrolling on the most popular screen resolution
8	Clear and consistent language	Keep consistency throughout with regard to layout and grammar
9	Font size	Avoid small text

If one can establish a reliable system for characterising the apps in terms of BCTs, engagement and ease-of-use features, then this methodology could be used to assess apps that have not been evaluated in RCTs. It could also provide a basis for characterising apps for which RCT evidence is available to identify potentially important components. With regard to assessing apps in the market, one could take a snapshot of the body of available apps at a given time with a view of making comparisons with apps developed in later years.

Thus, the main research question addressed by this study was can BCTs, engagement and ease-of-use features of smartphone apps to aid smoking cessation be identified reliably? A secondary question was what was the prevalence of these features in apps, which were available on the Apple app store in 2012?

## METHODS

### Study design

**Fig. 1:**
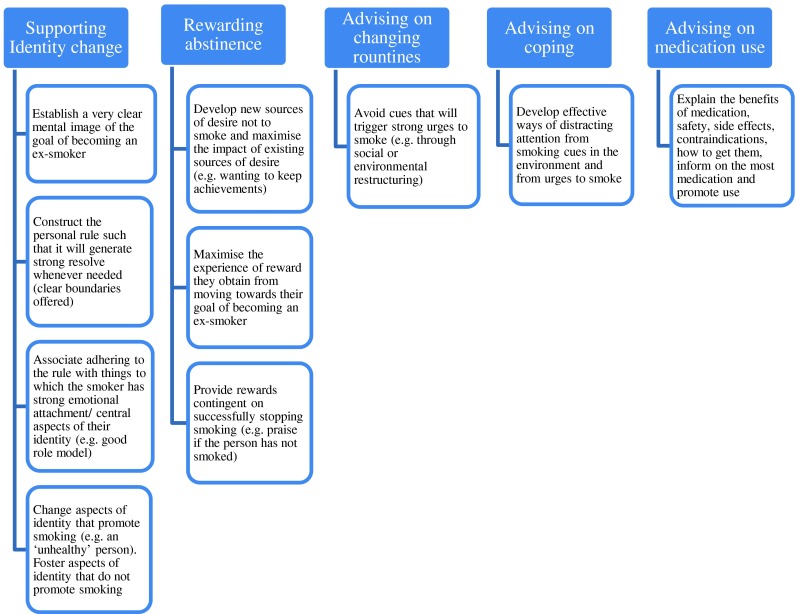
Framework for coding BCTs by function that are found to be positively associated with higher success rates for smoking cessation

Smoking cessation apps (both free and paid), available in English that purported to assist with smoking cessation, were searched on the Apple app store on 25 September 2012 (www.apple.com/itunes); 184 apps met the inclusion criteria and were downloaded (see supplementary file, Table A). The apps in this study were assessed on five specific BCTs that had been found previously to be associated with higher success in quitting smoking and can be implemented on a smartphone app without relying on an additional accessory. These BCTs were (1) strengthening ex-smoker identity, (2) providing rewards (usually praise) contingent on stopping successfully, (3) advising on changing routines, (4) advising and assisting with ways of coping with urges to smoke and (5) asking about use of stop-smoking medication and advising on use of stop-smoking medication (Fig. 1). From research literature and from discussions with app developers, a set of 11 features (Table 1) that could promote engagement and a set of nine features (Table 2) that could promote ease-of-use were identified.

### Coding of smoking cessation apps

Two coders independently assessed whether or not each of the five specific BCTs was present in each of the 184 apps. The coders were familiar with the English SSS as well as with the study and were given detailed instructions but were not given any further training. Approach of using just two coders has been a common practice in evaluating BCT taxonomies to arrive at an initial evaluation of the coding system [37]. The rationale for using two coders is that one is assessing the capacity in principle of the coding system to produce reliable results rather than evaluating the skills of groups of coders. BCTs were coded in a binary manner (present or absent) rather than on a quantitative scale because of difficulties in capturing the ‘amount’ or ‘quality’ of the BCT delivered. This approach is used widely [37–39]. There was no threshold for the number of times the BCT was used. One instance was enough for the BCT to be coded as present.

For features promoting engagement and ease-of-use, a slightly different method was used. Presence or absence of each of the 11 engagement and nine ease-of-use features was recorded, but given the large numbers of these features and the lack of evidence that any given feature would make a substantial difference by itself, we computed scores denoting the proportion of those that were adopted in each app. The scores can vary from 0 to 1 and can be interpreted as the proportion of features, which were actually judged to be present. Thus, if an app was recorded by a coder as possessing seven out of the available 11 engagement features, it received a score of 0.64 (i.e. 7/11). If an app possessed seven out of nine ease-of-use features, it received a score of 0.78 (i.e. 7/9). Multiplying this score by 100 would give a percentage of features that were present (for an example of how an app was coded using this approach, see supplementary file, Table B).

### Statistical analysis

The statistical analysis was performed using IBM SPSS statistics version 20.0. Inter-rater reliability was assessed by percentage agreement and by ‘prevalence and bias adjusted kappas’ (PABAK) [40] for BCTs. PABAK is considered preferable to kappa as it adjusts for potential chance agreement between coders and high prevalence of negative agreement (i.e. when both coders agree that the BCT is absent) [40]. For the interpretation of PABAK values, the following standard for strength of agreement was adopted [41] : <0.00 = poor, 0.00–0.20 = slight, 0.21–0.40 = fair, 0.41–0.60 = moderate, 0.61–0.80 = substantial and 0.81–1.00 = almost perfect agreement. A bias index was also calculated by reflecting different propensities of the two raters to code a BCT as present. A bias index close to zero indicates that the two coders judged approximately the same numbers of apps to have a given feature. A prevalence index was calculated by indexing the combined likelihood of coding a feature as present. A low prevalence index indicates a rare feature, while a high prevalence index indicates a feature that is judged to be commonly present. Intra-class correlation coefficients were calculated to assess level of agreement between coders in the scores for engagement and ease-of-use features. To calculate the prevalence estimates for BCTs, ‘lower’ and ‘upper’ estimates were made. The prevalence of lower estimates was based on both the coders identifying a BCT, while the prevalence of upper estimates was based on either of the two coders identifying a BCT. For the engagement and ease-of-use features, the scores of the two raters for each app were averaged.

## RESULTS

Percent agreement, PABAKs and bias and prevalence indices for the five specific BCTs are presented in Table 3. The percentage agreement for BCTs ranged from 66.8 % (advice on changing routines) to 95.1 % (advice on usage of stop-smoking medication). PABAK values ranged from 0.35 (advice on changing routines) to 0.90 (advice on usage of stop-smoking medication) (*p* < 0.001 in all cases). The intra-class correlation coefficients between the two coders for scores denoting the proportions of (a) a set of engagement features and (b) a set of ease-of-use features, which were included, were 0.77 and 0.75, respectively (*p* < 0.001).

**Table 3:** Percent agreement and PABAKs for specific BCTs that are found to be associated with higher success rates for smoking cessation

BCTs	Percentage agreement	Prevalence index	Bias index	PABAK
Supporting identity change	70.1	0.27	0.17	0.40
Rewarding abstinence	78.8	0.27	0.17	0.49
Advising on changing routines	66.8	−0.31	0.29	0.35
Advising on coping with cravings	69.6	−0.22	0.30	0.40
Advising on medication use	95.1	−0.88	0.05	0.90

*BCTs* behaviour change techniques, *PABAK* prevalence adjusted bias adjusted kappa

Based on the lower prevalence estimates, 89 apps (48.4 %) promoted an ex-smoker identity, 93 apps (50.5 %) provided contingent rewards (usually praise) on abstinence, 33 apps (17.9 %) advised on changing routines, and 44 apps (23.9 %) promoted techniques for coping with cravings, while seven apps (3.8 %) advised on using stop-smoking medication. Based on the upper prevalence estimates, 144 apps (78.3 %) promoted ex-smoker identity, 140 apps (76.1 %) provided contingent rewards (usually praise) on abstinence, 92 apps (50.0 %) advised on changing routines, and 99 apps (53.8 %) promoted techniques for coping with cravings, while 16 apps (8.7 %) advised on using stop-smoking medication (Table 4). The mean (SD) number of BCTs used was 1.45 (1.14) for lower and 2.67 (1.33) for upper estimates. The average proportions of specified engagement and ease-of-use features included in the apps were 69 and 83 %, respectively.

**Table 4:** Lower and upper prevalence estimates for specific BCTs in 184 smoking cessations apps

BCTs	Lower estimates	Upper estimates
Supporting identity change	48.4 %	78.3 %
Rewarding abstinence	50.5 %	76.1 %
Advising on changing routines	17.9 %	50.0 %
Advising on coping with cravings	23.9 %	53.8 %
Advising on medication use	3.8 %	8.7 %

*BCTs* behaviour change techniques

## DISCUSSION

This study found that the content of smoking cessation apps could be identified with fair to high reliability for inclusion of BCTs that are associated with higher success rates in face-to-face behavioural support for smoking cessation. The study also found that engagement and ease-of-use features could be identified with high reliability. Smoking cessation apps available on the Apple app store in 2012 tended to focus on supporting identity change and rewarding abstinence (usually praise), while a very few of those apps made reference to stop-smoking medication.

With a potentially reliable coding system, the opportunity now exists to assess associations between the identified features (BCTs, engagement and ease-of-use features) and app popularity, usage and effectiveness. The coding system could be used to characterise intervention apps and control apps in RCTs as well as other apps available in the market place. In principle, the coding system may also be extended to websites and to other digital interventions. This novel methodology can help identify specific features that would be expected to lead apps (and other digital interventions) to become more effective, engaging and easy to use.

As assessed by PABAK, the fact that some BCTs had a fair rather than high reliability indicates that there may be ways in which this coding methodology could be improved. When it comes to coding interventions using more generic BCT taxonomies, training programmes are available [22]. Similar training could potentially be made available for smoking-specific taxonomies. However, as found with other BCT coding schemes, the inter-rater reliability was considered moderate [38]. The high inter-rater reliability for engagement and ease-of-use features means that this coding scheme could provide a useful basis for assessing apps in the future.

The finding that advice on use of stop-smoking medication was rare is consistent with previous research [7, 8, 10]. However, there was one exception: A recently published study suggested that a reasonably large number of smoking cessation apps (41 out of 225 apps) assessed in the study discussed pharmaceutical products [11]. Based on the results from a majority of published studies, the advice on use of stop-smoking medication could be an important omission given that the use of, and adherence to, stop-smoking medication is typically low. Other research has found that adherence to smoking cessation medication was improved by means of a website-mediated intervention that increased cessation rates [42]. As found in other studies, use of other four BCTs was also relatively uncommon (lower estimates 17.9 to 50.5 %), which suggests that smoking cessation apps lack evidence-based content [7–11].

The fact that the apps, on an average, contained a relatively high proportion of engagement and ease-of-use features suggests that the designers were paying attention to issues such as high attrition rates. However, even if an app is engaging and easy to use does not necessarily mean that it will be effective for smoking cessation or for other health behaviour changes. For example, with fitness and weight loss apps, evidence suggests that popularity and usage are not related to effectiveness [43]. Scientific research into ‘health and fitness’ apps is at a very early stage, and this study is an initial but necessary step in the process of finding out how these apps should be designed for maximum effect.

### Limitations

One limitation of this study was that only Apple’s iPhone apps were evaluated. It seems unlikely that inclusion of Android apps would have substantially altered the assessment of coding reliability (as most of the apps are identical for iPhones as well as for Android phones). However, there may be some differences between apps available on iPhones and on Android phones in terms of design and functionality. A second limitation is that the coding only noted the presence or absence of BCTs, engagement and ease-of-use features. It is much more challenging to assess the extent of use of these features or their quality, even though both of these may play a major role in app effectiveness. This is an important area for future research. A third limitation is that while every effort was made to select relevant features, we may have missed some features that are crucial, in terms of content, effectiveness and engagement. For example, we did not code content features, which offered additional support or elicited users’ views. When the coding system is used to predict outcomes and actual engagement, it will be important to consider additional elements that might be relevant. A fourth limitation is that only two coders were used. This is a standard practice for BCT reliability studies. The coders were not selected to be experts and received little training and induction, it suggests that the system could be widely used. However, in future studies, it would be useful to examine whether a brief training curriculum provided to coders in using the smoking-specific taxonomy would maximise the reliability and confidence of using the taxonomy between coders. Furthermore, the inter-rater reliability was only moderate. This may be improved by putting a training programme in place. A fifth limitation is that the apps coded were the ones that were available in 2012. This is a rapidly developing space, and it will be interesting to see whether the developments in this area will lead future apps to become more evidence-based.

### Summary

In summary, this study found that it is possible to identify potentially useful BCTs in smoking cessation smartphone apps with fair to high inter-rater reliability and that it is possible to identify engagement and ease-of-use with high reliability. Smoking cessation apps available on the Apple app store in 2012 tended to focus on supporting identity change and rewarding abstinence (usually praise), while a very few of those apps made reference to stop-smoking medication.

## Electronic supplementary material

Below is the link to the electronic supplementary material.

ESM 1 (DOCX 96 kb)

ESM 2 (DOCX 348 kb)
