# Supplementary material for: Characterising smoking cessation smartphone applications in terms of behaviour change techniques, engagement and ease-of-use features
Source: Transl Behav Med. 2015 Nov 23;6(3):410–7. doi: 10.1007/s13142-015-0352-x (PMC4987605; doi:10.1007/s13142-015-0352-x)
Supplement: Supplementary file 1 — (DOCX 96 kb) [file 13142_2015_352_MOESM1_ESM.docx]

**Supplementary Section**

Table A: Names of the 184 smoking cessation apps that were reviewed and the BCTs present (lower and upper estimates), and the proportion of engagement features and ease-of use features used in these apps

| S.no. | Name of the Smoking Cessation App | Supporting identity change | Rewarding abstinence | Advising on changing routines | Advising on coping with cravings | Advising on medication use | Supporting identity change | Rewarding abstinence | Advising on changing routines | Advising on coping with cravings | Advising on medication use | Engagement Features | Ease-of-use Features |
| --- | --- | --- | --- | --- | --- | --- | --- | --- | --- | --- | --- | --- | --- |
|  |  | **Lower estimates** | | | | | **Upper estimates** | | | | |  |  |
| 1 | 20 minute deeply relaxing sleep with hypnosis | 0 | 0 | 1 | 0 | 0 | 0 | 0 | 1 | 0 | 0 | 82% | 89% |
| 2 | 20 minute deeply relaxing sleep with hypnosis | 0 | 0 | 1 | 0 | 0 | 0 | 0 | 1 | 0 | 0 | 82% | 89% |
| 3 | 6 self hypnosis courses | 0 | 0 | 0 | 0 | 0 | 1 | 1 | 0 | 0 | 0 | 36% | 83% |
| 4 | A breath of fresh air | 1 | 0 | 0 | 0 | 0 | 1 | 0 | 1 | 1 | 0 | 59% | 83% |
| 5 | Acupuncture treat yourself | 0 | 1 | 1 | 1 | 0 | 0 | 1 | 1 | 1 | 0 | 73% | 100% |
| 6 | Acupuncture treat yourself | 0 | 0 | 0 | 0 | 0 | 0 | 0 | 0 | 0 | 0 | 82% | 78% |
| 7 | Affirmation spell quit smoking magic | 0 | 0 | 0 | 0 | 0 | 0 | 0 | 0 | 0 | 0 | 45% | 89% |
| 8 | Anti resolutions 2010 | 1 | 0 | 1 | 0 | 0 | 1 | 0 | 1 | 0 | 0 | 91% | 100% |
| 9 | Anxiety free manage stress& feel better guided meditation, hypnosis subliminal | 0 | 0 | 0 | 0 | 0 | 0 | 0 | 0 | 0 | 0 | 64% | 78% |
| 10 | Bad habit | 0 | 1 | 0 | 0 | 0 | 1 | 1 | 1 | 0 | 0 | 77% | 94% |
| 11 | Biblical encouragement stop smoking | 1 | 1 | 0 | 0 | 0 | 1 | 1 | 0 | 0 | 0 | 77% | 78% |
| 12 | Break away | 0 | 1 | 0 | 0 | 0 | 1 | 1 | 0 | 0 | 0 | 64% | 67% |
| 13 | Buttoff | 0 | 0 | 0 | 0 | 0 | 1 | 1 | 1 | 1 | 0 | 64% | 94% |
| 14 | Cigarette emo counter | 0 | 1 | 0 | 0 | 0 | 1 | 1 | 0 | 0 | 0 | 77% | 94% |
| 15 | Daily | 0 | 1 | 0 | 0 | 0 | 1 | 1 | 0 | 0 | 0 | 64% | 94% |
| 16 | Day time affirmations on stopping smoking | 1 | 1 | 1 | 1 | 0 | 1 | 1 | 1 | 1 | 0 | 73% | 89% |
| 17 | Deep relaxation hypnosis subliminal guided meditation with store Erick Brown | 0 | 0 | 1 | 1 | 0 | 0 | 0 | 1 | 1 | 0 | 91% | 78% |
| 18 | Delux home screens and backgrounds | 0 | 0 | 0 | 0 | 0 | 1 | 0 | 0 | 0 | 0 | 59% | 89% |
| 19 | Easy Stop Smoking | 1 | 0 | 0 | 0 | 0 | 1 | 1 | 1 | 1 | 0 | 59% | 89% |
| 20 | eJuice | 1 | 0 | 0 | 0 | 0 | 1 | 0 | 0 | 0 | 0 | 41% | 44% |
| 21 | electric smoke | 0 | 1 | 0 | 0 | 0 | 1 | 1 | 1 | 1 | 0 | 77% | 83% |
| 22 | eQuit Alert | 1 | 0 | 0 | 0 | 0 | 1 | 1 | 0 | 0 | 0 | 68% | 100% |
| 23 | esense stop smoking guide | 0 | 0 | 0 | 0 | 1 | 1 | 1 | 1 | 1 | 1 | 45% | 83% |
| 24 | Ether | 0 | 0 | 0 | 0 | 0 | 0 | 0 | 0 | 0 | 0 | 36% | 22% |
| 25 | Fat burner self hypnosis Erick Brown | 0 | 0 | 1 | 1 | 0 | 0 | 0 | 1 | 1 | 0 | 91% | 78% |
| 26 | Fitness and Exercise Motivation Hypnosis by Erick Brown | 0 | 0 | 1 | 1 | 0 | 1 | 0 | 1 | 1 | 0 | 91% | 78% |
| 27 | Focus & concentration self hypnosis Erick Brown | 0 | 0 | 1 | 1 | 0 | 0 | 0 | 1 | 1 | 0 | 91% | 78% |
|  |  | **Lower estimates** | | | | | **Upper estimates** | | | | |  |  |
| 28 | Free hypnosis | 0 | 0 | 0 | 0 | 0 | 1 | 1 | 0 | 0 | 0 | 23% | 39% |
| 29 | Free yourself from fear | 1 | 0 | 1 | 1 | 0 | 1 | 0 | 1 | 1 | 0 | 36% | 33% |
| 30 | Good Resolutions | 0 | 1 | 0 | 0 | 0 | 1 | 1 | 1 | 0 | 0 | 82% | 100% |
| 31 | Good resolutions 2010 | 0 | 1 | 0 | 0 | 0 | 1 | 1 | 1 | 0 | 0 | 82% | 100% |
| 32 | Gotta kickit now | 1 | 1 | 0 | 0 | 0 | 1 | 1 | 0 | 0 | 0 | 73% | 94% |
| 33 | Green lungs quit smoking | 1 | 1 | 0 | 0 | 0 | 1 | 1 | 0 | 0 | 0 | 73% | 89% |
| 34 | H4UTobacco | 0 | 0 | 0 | 0 | 0 | 1 | 0 | 0 | 0 | 0 | 45% | 89% |
| 35 | Habit Ninja | 0 | 1 | 0 | 0 | 0 | 1 | 1 | 0 | 0 | 0 | 73% | 94% |
| 36 | Help I want to give up smoking | 1 | 0 | 0 | 0 | 0 | 1 | 1 | 1 | 1 | 0 | 36% | 56% |
| 37 | How to never smoke again by Alex Dollery | 1 | 0 | 0 | 0 | 0 | 1 | 1 | 1 | 1 | 0 | 45% | 61% |
| 38 | How to quit smoking | 0 | 0 | 0 | 0 | 0 | 1 | 1 | 1 | 1 | 0 | 45% | 67% |
| 39 | How to stop smoking in 1 week | 1 | 0 | 0 | 0 | 0 | 1 | 1 | 1 | 1 | 1 | 64% | 61% |
| 40 | HPB iQuit | 1 | 1 | 1 | 1 | 0 | 1 | 1 | 1 | 1 | 0 | 95% | 89% |
| 41 | Hypnomatic | 1 | 0 | 0 | 0 | 0 | 1 | 0 | 0 | 0 | 0 | 64% | 33% |
| 42 | Hypnoquit - stop smoking with Diana Parkinson | 0 | 0 | 0 | 0 | 0 | 1 | 1 | 1 | 0 | 0 | 55% | 94% |
| 43 | Hypnosis direct stop smoking | 0 | 0 | 0 | 0 | 0 | 1 | 1 | 1 | 1 | 0 | 45% | 89% |
| 44 | Hypnosis Lessons | 0 | 0 | 1 | 0 | 0 | 0 | 0 | 1 | 0 | 0 | 45% | 78% |
| 45 | Hypnosis light guided mediation subliminal Audio | 0 | 0 | 1 | 1 | 0 | 0 | 0 | 1 | 1 | 0 | 73% | 100% |
| 46 | Hypnosis motivation for weight loss & self esteem- subliminal guided meditation Erick Brown | 0 | 0 | 1 | 1 | 0 | 0 | 0 | 1 | 1 | 0 | 91% | 78% |
| 47 | Hypnosis secrets | 0 | 0 | 1 | 1 | 0 | 0 | 0 | 1 | 1 | 0 | 55% | 89% |
| 48 | Hypnosis sleep soundly | 0 | 0 | 1 | 0 | 0 | 0 | 0 | 1 | 0 | 0 | 55% | 78% |
| 49 | Hypnosis sleep soundly | 0 | 0 | 0 | 0 | 0 | 0 | 0 | 0 | 0 | 0 | 64% | 44% |
| 50 | Hypnosis sleep well deeper sleep to better sleep Erick Brown | 0 | 0 | 1 | 1 | 0 | 0 | 0 | 1 | 1 | 0 | 91% | 78% |
| 51 | Hypnotherapy news | 0 | 0 | 0 | 0 | 0 | 0 | 0 | 0 | 0 | 0 | 36% | 67% |
| 52 | iCan Stop Smoking | 1 | 0 | 0 | 0 | 0 | 1 | 1 | 1 | 1 | 0 | 55% | 83% |
| 53 | iQSmoking | 1 | 1 | 0 | 1 | 0 | 1 | 1 | 0 | 1 | 0 | 82% | 94% |
| 54 | iQuit | 1 | 1 | 0 | 0 | 0 | 1 | 1 | 0 | 0 | 0 | 77% | 94% |
| 55 | iQuit - Stop Smoking Counter | 1 | 1 | 0 | 1 | 0 | 1 | 1 | 0 | 1 | 1 | 100% | 100% |
| 56 | iQuit smoking | 0 | 0 | 0 | 0 | 0 | 1 | 1 | 1 | 1 | 0 | 45% | 78% |
| 57 | iQuit smoking graph tracking | 1 | 1 | 0 | 0 | 0 | 1 | 1 | 0 | 0 | 0 | 77% | 94% |
| 58 | Iquit smoking how to stop | 0 | 0 | 0 | 0 | 0 | 1 | 1 | 0 | 0 | 0 | 64% | 94% |
| 59 | iSmoke2Much | 0 | 1 | 0 | 1 | 0 | 0 | 1 | 0 | 1 | 0 | 64% | 89% |
| 60 | istopsmoking | 1 | 1 | 0 | 1 | 0 | 1 | 1 | 0 | 1 | 0 | 82% | 100% |
| 61 | istopsmoking | 1 | 1 | 0 | 0 | 0 | 1 | 1 | 0 | 0 | 0 | 73% | 94% |
|  |  | **Lower estimates** | | | | | **Upper estimates** | | | | |  |  |
| 62 | Kick the habit | 0 | 1 | 0 | 0 | 0 | 1 | 1 | 1 | 1 | 0 | 68% | 94% |
| 63 | Kickit Quit smoking incentivised | 1 | 1 | 0 | 0 | 0 | 1 | 1 | 0 | 0 | 0 | 77% | 94% |
| 64 | Kwit | 1 | 1 | 0 | 1 | 0 | 1 | 1 | 0 | 1 | 0 | 91% | 100% |
| 65 | Kwit - Quit Smoking is a Game | 1 | 1 | 0 | 1 | 0 | 1 | 1 | 0 | 1 | 0 | 91% | 10% |
| 66 | Love not smoking do something different | 1 | 1 | 0 | 0 | 0 | 1 | 1 | 1 | 1 | 0 | 95% | 94% |
| 67 | Lungs check no smoking | 0 | 1 | 0 | 0 | 0 | 1 | 1 | 0 | 0 | 0 | 73% | 89% |
| 68 | Make money with the law of attraction Erick Brown | 0 | 0 | 1 | 1 | 0 | 0 | 0 | 1 | 1 | 0 | 91% | 78% |
| 69 | Motivator stop smoking with your personal motivator | 1 | 1 | 0 | 0 | 0 | 1 | 1 | 0 | 0 | 0 | 82% | 94% |
| 70 | My Health Coach - Stop Smoking with Alan Carr | 1 | 1 | 0 | 0 | 0 | 1 | 1 | 1 | 1 | 0 | 82% | 89% |
| 71 | My hypnosis stop smoking lite version | 1 | 0 | 0 | 0 | 0 | 1 | 1 | 1 | 1 | 0 | 68% | 94% |
| 72 | My Last Cigarette - Stop smoking stay quit | 1 | 1 | 0 | 1 | 1 | 1 | 1 | 0 | 1 | 1 | 91% | 67% |
| 73 | My Last Cigarette - Stop smoking stay quit | 1 | 1 | 0 | 0 | 0 | 1 | 1 | 0 | 0 | 0 | 86% | 89% |
| 74 | My mobile guru | 0 | 0 | 0 | 0 | 0 | 1 | 0 | 0 | 0 | 0 | 23% | 39% |
| 75 | My Quitting Math | 1 | 1 | 0 | 1 | 0 | 1 | 1 | 0 | 1 | 0 | 73% | 100% |
| 76 | My smoking Log | 0 | 1 | 0 | 0 | 0 | 0 | 1 | 0 | 0 | 0 | 68% | 94% |
| 77 | My stop buddy quit smoking | 1 | 1 | 0 | 0 | 0 | 1 | 1 | 1 | 1 | 0 | 82% | 94% |
| 78 | Myquit time | 1 | 1 | 0 | 0 | 0 | 1 | 1 | 0 | 1 | 0 | 77% | 94% |
| 79 | NHS Quit Smoking | 1 | 1 | 0 | 1 | 0 | 1 | 1 | 1 | 1 | 1 | 77% | 83% |
| 80 | Nicorette active stop | 1 | 1 | 0 | 0 | 0 | 1 | 1 | 1 | 1 | 1 | 82% | 94% |
| 81 | Nicot Lite | 0 | 1 | 0 | 0 | 0 | 1 | 1 | 0 | 0 | 0 | 55% | 61% |
| 82 | Nicotine gum timer | 1 | 0 | 0 | 0 | 0 | 1 | 1 | 0 | 0 | 0 | 73% | 100% |
| 83 | No smoke | 1 | 0 | 0 | 0 | 0 | 1 | 1 | 0 | 0 | 0 | 27% | 67% |
| 84 | No Smoking | 0 | 1 | 0 | 0 | 0 | 1 | 1 | 1 | 1 | 1 | 77% | 94% |
| 85 | No smoking | 0 | 1 | 0 | 0 | 0 | 0 | 1 | 0 | 0 | 0 | 68% | 94% |
| 86 | No Smoking Game | 0 | 0 | 0 | 0 | 0 | 0 | 0 | 0 | 0 | 0 | 45% | 67% |
| 87 | No smoking Life | 1 | 1 | 0 | 0 | 0 | 1 | 1 | 0 | 0 | 0 | 64% | 83% |
| 88 | Non smoker maker Barbara Ford Hammond | 1 | 0 | 0 | 0 | 0 | 1 | 1 | 1 | 1 | 0 | 55% | 89% |
| 89 | Non smoker motivation | 1 | 1 | 0 | 0 | 0 | 1 | 1 | 0 | 0 | 0 | 77% | 94% |
| 90 | One less | 0 | 0 | 0 | 0 | 0 | 0 | 1 | 0 | 0 | 0 | 55% | 94% |
| 91 | Pass the ugly stick | 0 | 0 | 0 | 0 | 0 | 1 | 1 | 0 | 0 | 0 | 73% | 89% |
| 92 | Past Life regression Erick Brown | 0 | 0 | 1 | 1 | 0 | 0 | 0 | 1 | 1 | 0 | 91% | 78% |
| 93 | Q smoking | 1 | 0 | 0 | 0 | 0 | 1 | 0 | 0 | 0 | 0 | 73% | 78% |
| 94 | Quit Advisor | 1 | 0 | 0 | 0 | 0 | 1 | 1 | 0 | 0 | 0 | 59% | 61% |
| 95 | Quit And Be Free | 1 | 1 | 1 | 1 | 1 | 1 | 1 | 1 | 1 | 1 | 86% | 100% |
|  |  | **Lower estimates** | | | | | **Upper estimates** | | | | |  |  |
| 96 | Quit Charge | 1 | 1 | 1 | 0 | 0 | 1 | 1 | 1 | 0 | 0 | 73% | 94% |
| 97 | Quit for life programme stop smoking urge tracker | 0 | 1 | 0 | 0 | 0 | 1 | 1 | 1 | 1 | 1 | 68% | 89% |
| 98 | Quit forever | 1 | 1 | 1 | 0 | 1 | 1 | 1 | 1 | 1 | 1 | 77% | 72% |
| 99 | Quit It App | 0 | 0 | 0 | 1 | 0 | 1 | 1 | 1 | 1 | 0 | 77% | 89% |
| 100 | Quit it light stop smoking now | 1 | 1 | 0 | 1 | 0 | 1 | 1 | 0 | 1 | 0 | 82% | 100% |
| 101 | Quit it Stop Smoking | 1 | 1 | 0 | 0 | 0 | 1 | 1 | 0 | 0 | 0 | 82% | 100% |
| 102 | Quit Now! | 1 | 1 | 0 | 0 | 0 | 1 | 1 | 1 | 0 | 0 | 86% | 89% |
| 103 | quit smokign stop smoking hypnosis programme | 0 | 0 | 0 | 0 | 0 | 1 | 1 | 1 | 1 | 0 | 41% | 83% |
| 104 | Quit Smoking | 1 | 1 | 0 | 0 | 0 | 1 | 1 | 0 | 0 | 0 | 68% | 94% |
| 105 | Quit smoking 1 week + bonus | 1 | 0 | 0 | 0 | 0 | 1 | 1 | 1 | 1 | 0 | 45% | 56% |
| 106 | Quit smoking assistant | 1 | 1 | 0 | 0 | 0 | 1 | 1 | 1 | 1 | 0 | 77% | 100% |
| 107 | Quit smoking coach | 1 | 1 | 1 | 1 | 1 | 1 | 1 | 1 | 1 | 1 | 82% | 89% |
| 108 | Quit smoking cold turkey | 1 | 1 | 0 | 0 | 0 | 1 | 1 | 1 | 1 | 0 | 86% | 94% |
| 109 | Quit smoking for good | 0 | 0 | 0 | 0 | 0 | 1 | 1 | 1 | 1 | 0 | 41% | 50% |
| 110 | Quit smoking for life | 1 | 1 | 0 | 0 | 0 | 1 | 1 | 1 | 1 | 0 | 82% | 78% |
| 111 | Quit smoking helper | 0 | 1 | 0 | 0 | 0 | 1 | 1 | 0 | 0 | 0 | 86% | 89% |
| 112 | Quit Smoking in 5 Days | 1 | 0 | 1 | 1 | 0 | 1 | 0 | 1 | 1 | 0 | 86% | 89% |
| 113 | Quit smoking manager | 0 | 1 | 0 | 0 | 0 | 0 | 1 | 0 | 0 | 0 | 73% | 94% |
| 114 | Quit smoking now | 0 | 1 | 0 | 0 | 0 | 1 | 1 | 1 | 1 | 0 | 45% | 94% |
| 115 | Quit Smoking Now with Max Kirsten | 1 | 1 | 0 | 0 | 0 | 1 | 1 | 1 | 1 | 0 | 77% | 78% |
| 116 | Quit smoking test | 0 | 1 | 0 | 0 | 0 | 1 | 1 | 0 | 0 | 0 | 68% | 89% |
| 117 | Quit smoking the easy way | 1 | 0 | 0 | 0 | 0 | 1 | 1 | 1 | 1 | 1 | 45% | 56% |
| 118 | quit smoking, stop smoking, hypnosis plan | 1 | 0 | 0 | 0 | 0 | 1 | 0 | 0 | 0 | 0 | 77% | 72% |
| 119 | Quitnowpro | 0 | 1 | 1 | 0 | 0 | 0 | 1 | 1 | 0 | 0 | 73% | 89% |
| 120 | Quitsmoking pro | 1 | 1 | 0 | 1 | 0 | 1 | 1 | 0 | 1 | 0 | 91% | 78% |
| 121 | QuitSTART | 1 | 1 | 1 | 1 | 0 | 1 | 1 | 1 | 1 | 0 | 91% | 89% |
| 122 | Quitter | 0 | 1 | 0 | 0 | 0 | 1 | 1 | 0 | 0 | 0 | 82% | 78% |
| 123 | Relax & sleep well by Glenn Harrold - A relaxation self hypnosis meditation | 0 | 0 | 0 | 0 | 0 | 1 | 0 | 0 | 0 | 0 | 27% | 39% |
| 124 | Say <<no>> to smoking | 0 | 0 | 0 | 0 | 0 | 0 | 0 | 0 | 0 | 0 | 59% | 72% |
| 125 | Say no to smoking | 0 | 0 | 0 | 0 | 0 | 1 | 1 | 0 | 0 | 0 | 59% | 83% |
| 126 | Self Hypnosis Downloads | 0 | 0 | 1 | 0 | 0 | 0 | 0 | 1 | 0 | 0 | 45% | 78% |
| 127 | Shocking Smoking facts | 0 | 0 | 0 | 0 | 0 | 1 | 1 | 0 | 0 | 0 | 59% | 83% |
| 128 | Sick of smoking | 1 | 1 | 0 | 0 | 0 | 1 | 1 | 1 | 1 | 0 | 77% | 94% |
| 129 | Since iQuit | 0 | 1 | 0 | 0 | 0 | 1 | 1 | 0 | 0 | 0 | 77% | 89% |
| 130 | Smoke diary | 0 | 0 | 0 | 0 | 0 | 1 | 1 | 0 | 0 | 0 | 77% | 94% |
| 131 | Smoke out | 1 | 1 | 0 | 0 | 0 | 1 | 1 | 0 | 0 | 0 | 68% | 94% |
| 132 | Smokebook | 0 | 1 | 0 | 0 | 0 | 0 | 1 | 0 | 0 | 0 | 68% | 100% |
|  |  | **Lower estimates** | | | | | **Upper estimates** | | | | |  |  |
| 133 | Smokefree fv | 0 | 1 | 0 | 0 | 0 | 1 | 1 | 0 | 0 | 0 | 77% | 94% |
| 134 | Smokefree lite | 0 | 1 | 0 | 0 | 0 | 1 | 1 | 1 | 1 | 0 | 77% | 89% |
| 135 | SF28 (SmokeFree28) | 1 | 1 | 1 | 1 | 1 | 1 | 1 | 1 | 1 | 1 | 86% | 89% |
| 136 | Smokeless Quit smoking | 0 | 1 | 0 | 0 | 0 | 1 | 1 | 0 | 1 | 0 | 73% | 100% |
| 137 | Smokeout | 1 | 1 | 0 | 1 | 0 | 1 | 1 | 0 | 1 | 0 | 82% | 89% |
| 138 | Smokfreespanish | 1 | 1 | 0 | 1 | 0 | 1 | 1 | 0 | 1 | 0 | 82% | 100% |
| 139 | Smoking horrors | 0 | 0 | 0 | 0 | 0 | 0 | 1 | 0 | 0 | 0 | 55% | 72% |
| 140 | Smoking Management | 1 | 1 | 0 | 0 | 0 | 1 | 1 | 0 | 0 | 0 | 68% | 89% |
| 141 | Smoktivation: My motivation to quit smoking | 1 | 1 | 0 | 1 | 0 | 1 | 1 | 0 | 1 | 0 | 91% | 89% |
| 142 | So You Think You Can Quit | 1 | 0 | 0 | 0 | 0 | 1 | 1 | 1 | 0 | 1 | 77% | 78% |
| 143 | Stop cigarettes | 0 | 0 | 0 | 0 | 0 | 0 | 1 | 0 | 0 | 0 | 68% | 94% |
| 144 | Stop smoke | 0 | 0 | 0 | 0 | 0 | 0 | 1 | 0 | 0 | 0 | 59% | 89% |
| 145 | Stop smoking | 0 | 1 | 0 | 0 | 0 | 1 | 1 | 0 | 1 | 0 | 41% | 67% |
| 146 | Stop smoking | 0 | 0 | 0 | 0 | 0 | 1 | 0 | 0 | 0 | 0 | 50% | 89% |
| 147 | Stop smoking audio books | 0 | 0 | 0 | 0 | 0 | 1 | 1 | 1 | 1 | 0 | 41% | 72% |
| 148 | Stop smoking coach | 0 | 0 | 0 | 0 | 0 | 1 | 1 | 0 | 1 | 0 | 41% | 50% |
| 149 | Stop smoking Dawn Grant | 1 | 0 | 0 | 0 | 0 | 1 | 1 | 1 | 1 | 0 | 73% | 61% |
| 150 | Stop Smoking for iphone | 0 | 0 | 0 | 0 | 0 | 1 | 1 | 0 | 0 | 0 | 64% | 89% |
| 151 | Stop smoking forever hypnosis by Glenn Harrold | 1 | 0 | 0 | 0 | 0 | 1 | 1 | 1 | 1 | 0 | 64% | 78% |
| 152 | Stop smoking full | 1 | 0 | 0 | 0 | 0 | 1 | 1 | 0 | 1 | 0 | 82% | 89% |
| 153 | Stop Smoking Hypnosis and Affirmations Program | 0 | 0 | 1 | 1 | 0 | 0 | 0 | 1 | 1 | 0 | 73% | 100% |
| 154 | Stop Smoking in 2 hours | 1 | 0 | 0 | 0 | 0 | 1 | 1 | 1 | 1 | 0 | 64% | 83% |
| 155 | Stop smoking in 5 days | 1 | 1 | 0 | 1 | 0 | 1 | 1 | 1 | 1 | 0 | 82% | 83% |
| 156 | Stop Smoking in 5 days - Leopard Edition | 1 | 1 | 0 | 0 | 0 | 1 | 1 | 1 | 1 | 0 | 73% | 89% |
| 157 | stop smoking in one hour with valerie austin | 0 | 0 | 0 | 0 | 0 | 1 | 1 | 1 | 1 | 0 | 50% | 50% |
| 158 | Stop smoking lite | 1 | 0 | 0 | 0 | 0 | 1 | 1 | 1 | 1 | 0 | 82% | 94% |
| 159 | Stop Smoking Manager SMO Quit | 1 | 1 | 0 | 1 | 0 | 1 | 1 | 0 | 1 | 0 | 82% | 89% |
| 160 | Stop smoking now cardio workout affirmations | 1 | 1 | 1 | 1 | 0 | 1 | 1 | 1 | 1 | 0 | 73% | 89% |
| 161 | Stop Smoking now Pocket hypnotherapy | 1 | 0 | 0 | 0 | 0 | 1 | 1 | 1 | 1 | 0 | 59% | 83% |
| 162 | Stop smoking now! | 1 | 1 | 0 | 0 | 0 | 1 | 1 | 1 | 1 | 0 | 77% | 78% |
| 163 | Stop Smoking Pro | 1 | 1 | 0 | 1 | 0 | 1 | 1 | 0 | 1 | 0 | 91% | 78% |
| 164 | stop smoking pro | 1 | 1 | 0 | 0 | 0 | 1 | 1 | 1 | 1 | 0 | 86% | 94% |
| 165 | Stop Smoking Quit nicotine addiction (Smoke Free) | 0 | 0 | 0 | 0 | 0 | 1 | 0 | 0 | 0 | 0 | 64% | 94% |
| 166 | Stop smoking timer | 0 | 1 | 0 | 0 | 0 | 1 | 1 | 0 | 0 | 0 | 73% | 94% |
|  |  | **Lower estimates** | | | | | **Upper estimates** | | | | |  |  |
| 167 | Stop smoking with hypnosis - Benjamin Bonnetti | 0 | 1 | 0 | 0 | 0 | 1 | 1 | 1 | 0 | 0 | 59% | 78% |
| 168 | stop smoking with self hypnosis with erick brown | 1 | 0 | 0 | 0 | 0 | 1 | 1 | 1 | 1 | 0 | 68% | 89% |
| 169 | Stop smoking! | 0 | 0 | 0 | 0 | 0 | 0 | 0 | 0 | 0 | 0 | 59% | 50% |
| 170 | Stop!+ | 0 | 1 | 0 | 0 | 0 | 1 | 1 | 0 | 0 | 0 | 73% | 94% |
| 171 | Stopsmoking in 2hours | 1 | 1 | 0 | 1 | 0 | 1 | 1 | 0 | 1 | 0 | 82% | 89% |
| 172 | The Best No Smoking App | 1 | 1 | 0 | 1 | 0 | 1 | 1 | 0 | 1 | 0 | 82% | 78% |
| 173 | The best no smoking app | 1 | 1 | 0 | 0 | 0 | 1 | 1 | 1 | 1 | 0 | 77% | 94% |
| 174 | The joy of quit smoking lite | 1 | 1 | 0 | 0 | 0 | 1 | 1 | 1 | 1 | 0 | 77% | 94% |
| 175 | The painless stop smoking cure | 0 | 1 | 0 | 0 | 0 | 1 | 1 | 1 | 1 | 0 | 77% | 94% |
| 176 | The power of positive thinking Erick Brown | 0 | 0 | 1 | 1 | 0 | 0 | 0 | 1 | 1 | 0 | 91% | 78% |
| 177 | The talking heart | 0 | 0 | 0 | 0 | 0 | 0 | 0 | 0 | 0 | 0 | 45% | 83% |
| 178 | Three Min Start Quit Smoking | 0 | 0 | 1 | 1 | 0 | 0 | 0 | 1 | 1 | 0 | 77% | 83% |
| 179 | Tobacco quitter app bundle | 0 | 0 | 0 | 0 | 0 | 1 | 1 | 1 | 1 | 1 | 77% | 94% |
| 180 | Tobacco tracker | 0 | 1 | 0 | 0 | 0 | 0 | 1 | 0 | 0 | 0 | 64% | 94% |
| 181 | Top 10 ways to quit smoking | 0 | 0 | 0 | 0 | 1 | 0 | 0 | 0 | 0 | 1 | 45% | 61% |
| 182 | Tyzen the Hypnotist - Audio Hypnotherapy to Lose Weight Beat Stress Quit Smoking & Attract Women | 0 | 0 | 1 | 1 | 0 | 1 | 0 | 1 | 1 | 0 | 64% | 83% |
| 183 | UCSF/SFGH Stop smoking | 1 | 1 | 0 | 0 | 0 | 1 | 1 | 1 | 1 | 0 | 82% | 94% |
| 184 | You can quit smoking | 1 | 0 | 0 | 0 | 0 | 1 | 1 | 1 | 1 | 0 | 36% | 50% |
