# Supplementary material for: Characterising smoking cessation smartphone applications in terms of behaviour change techniques, engagement and ease-of-use features
Source: Transl Behav Med. 2015 Nov 23;6(3):410–7. doi: 10.1007/s13142-015-0352-x (PMC4987605; doi:10.1007/s13142-015-0352-x)
Supplement: Supplementary file 2 — (DOCX 348 kb) [file 13142_2015_352_MOESM2_ESM.docx]

Table B: An example of how an app was coded based on the five specific BCTs, engagement and ease-of-use features

| Name of the smoking cessation app:SF28(SmokeFree28)  Feature | | Score (1=BCT present, 0=absent) | Rationale for the score |
| --- | --- | --- | --- |
| BCT 1 | Supporting identity change | 1 | 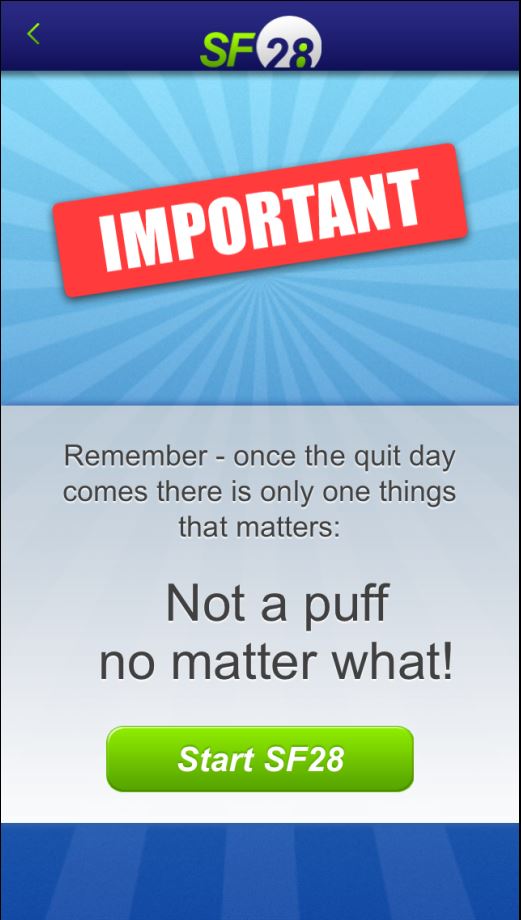  **App supporting identity change** e.g. app establishing a very clear mental image of the goal of becoming an ex-smoker |
| BCT 2 | Rewarding abstinence (usually praise) | 1 | 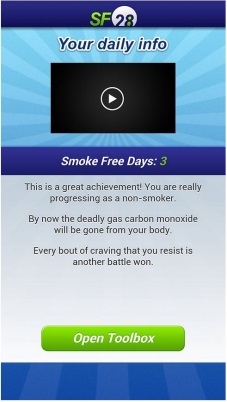  **App rewarding abstinence** e.g. praising the user for not smoking |
| BCT 3 | Advising on changing routines | 1 | 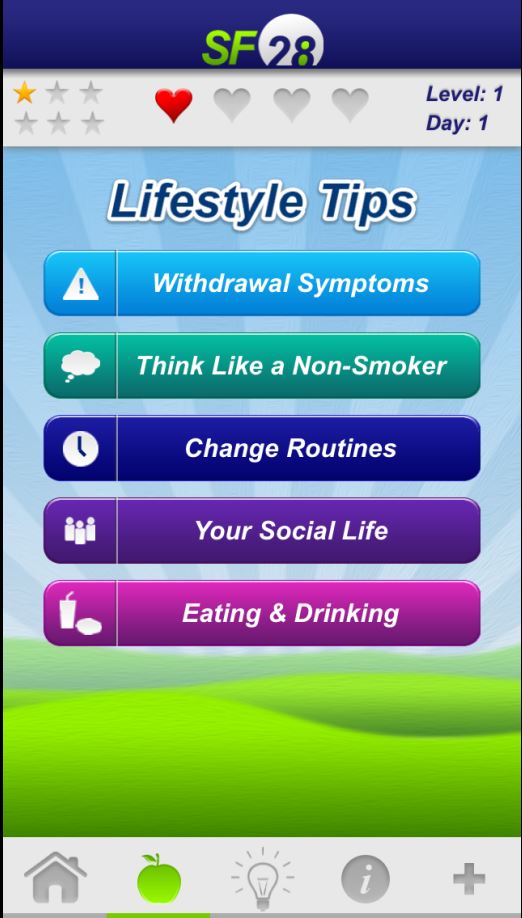  **App advising on changing routines** e.g. app providing various lifestyle tips on ways to change routines to minimise exposure to smoking cues |
| BCT 4 | Advising on coping with cravings | 1 | 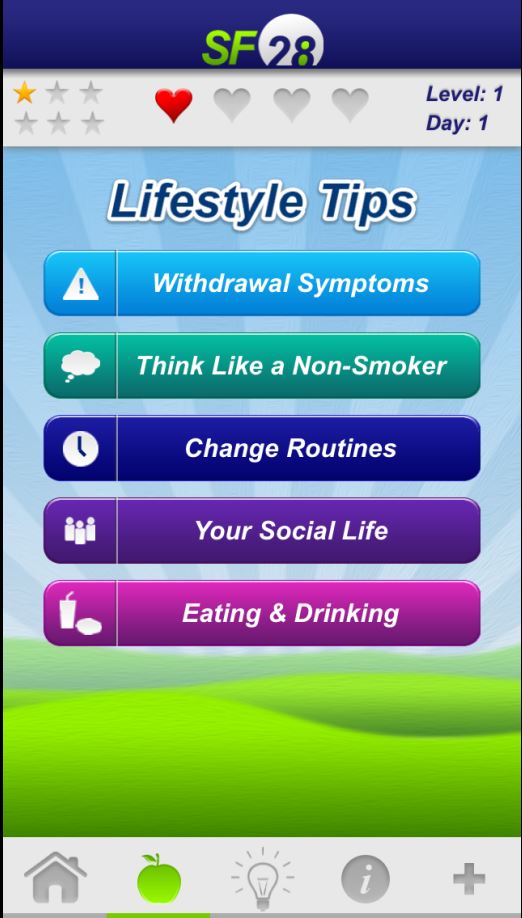  **App advising on coping with cravings** e.g. app providing various lifestyle tips on ways to cope with cravings |
| BCT 5 | Advising on stop-smoking medication use | 1 | 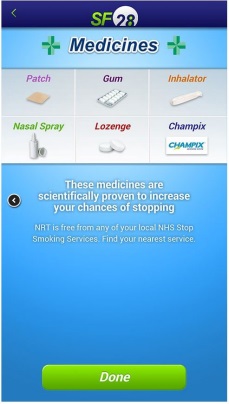  **App advising on use of stop-smoking medication** e.g. app explaining the benefits of medication and how to use them effectively |
| Engagement features | 11 engagement features | Average proportion of specified engagement features identified in the SF28 app: 86% | 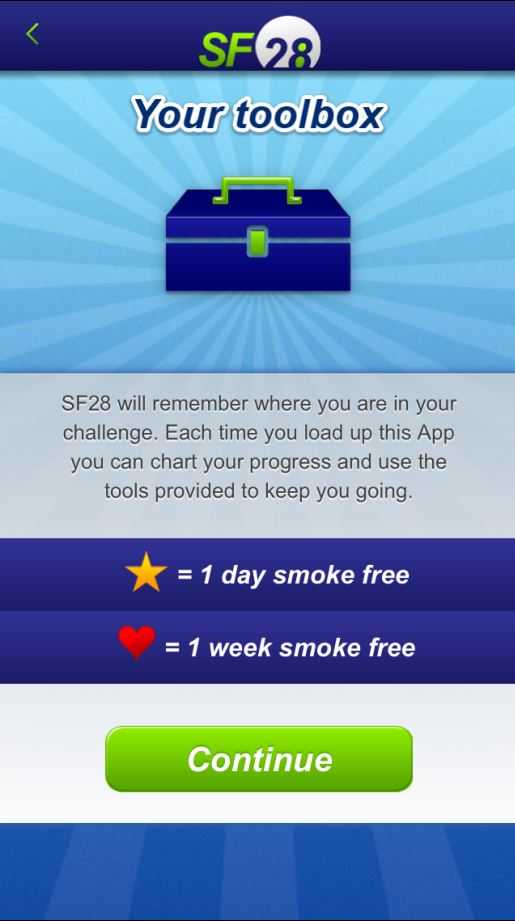  **Some of the engagement features can be seen on the screen** e.g. gamification concepts are used to engage users (such as, each consecutive day of abstinence was ‘rewarded’ by the app with the addition of a star on the home screen and every week with a heart) |
| ease-of-use features | 9 ease-of-use features | Average proportion of specified ease-of-use features identified in the SF28 app:  89% | 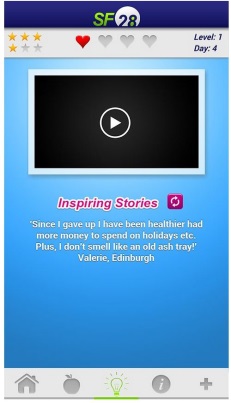  **Some of the ease-of-use features can be seen on the screen** e.g. page is named clearly ‘inspiring stories’ for a consistent and straightforward navigation, information presented on the page is clear and easy to read |
